# A systematic review of the relationship between neighborhood stressors, discrimination, and cardiometabolic outcomes during pregnancy

**DOI:** 10.1038/s44294-025-00072-0

**Published:** 2025-04-25

**Authors:** Roma Dhingra, Kosuke Tamura, Jinani Jayasekera, Amina P. Alio, Allana T. Forde

**Affiliations:** 1 Division of Intramural Research, National Institute on Minority Health and Health Disparities, National Institutes of Health, Bethesda, MD USA; 2 Department of Public Health Sciences, University of Rochester Medical Center, Rochester, NY USA

**Keywords:** Diseases, Health care

## Abstract

Cardiometabolic outcomes during pregnancy, including hypertensive disorders of pregnancy (HDP) and gestational diabetes, disproportionately affect racial and/or ethnic minority groups in the United States. These disparities are not fully explained by traditional risk factors, but race-related psychosocial stressors such as perceived neighborhood stressors and discrimination (PNSD) may contribute to adverse health outcomes. This systematic review examined the literature on the impact of PNSD on HDP and gestational diabetes. A comprehensive search of PubMed, PsycINFO, Embase, Web of Science, and CINAHL identified 10 eligible studies: seven cohort and three cross-sectional studies. Five studies reported significant associations between PNSD and increased risk for cardiometabolic outcomes during pregnancy (HDP-1 study, gestational diabetes-3 studies, both hypertension and diabetes during pregnancy-1 study). The included studies demonstrated good methodological quality. These findings suggest that PNSD may be associated with cardiometabolic outcomes during pregnancy, but further research is needed, particularly on perceived neighborhood stressors.

## Introduction

Hypertensive disorders of pregnancy and gestational diabetes are two of the most common pregnancy complications and pose significant risks to long-term maternal health^1,2^. Hypertensive disorders of pregnancy include a group of conditions (e.g., preeclampsia, gestational hypertension) characterized by high blood pressure that arises during pregnancy^3^. Gestational diabetes is defined by hyperglycemia that develops during pregnancy^4^. Pregnant women who belong to a racial and/or ethnic minority group have disproportionately higher incidence rates of hypertensive disorders of pregnancy and gestational diabetes^5^. Gestational diabetes in the United States (US) is most prevalent among Asian women (14.9%), followed by Hispanic women (8.5%)^6^. Additionally, Black and Hispanic women have the highest prevalence of hypertensive disorders of pregnancy^7,8^, and among women with hypertensive disorders of pregnancy, Black and Hispanic women have a higher risk of developing cardiovascular disease (CVD) after pregnancy compared to their White counterparts^8^. Therefore, exposures that are more common among racial and/or ethnic minority groups may represent potential modifiable causes for these racial and/or ethnic disparities.

Race-related psychosocial exposures are psychosocial stressors that are more prevalent among socially stigmatized racial and/or ethnic groups^9^. Structural discrimination refers to discriminatory institutions and policies such as racial residential segregation and mortgage discrimination, which result in inferior built environments (e.g., concentrated poverty within neighborhoods, substandard housing quality, fewer public amenities) in neighborhoods where racially marginalized groups mostly reside^10,11^. According to the health equity framework for social determinants of health (SDOH), structural discrimination shapes neighborhood built environments and social contexts, which can then influence lived personal experiences and contribute to perceived neighborhood stress^10,12^. Specifically, individuals may experience distress in response to physical attributes of their neighborhood, such as worrying about their safety while living in neighborhoods with higher crime rates^13^. Chronic psychosocial stressors can then contribute to adverse physiological responses such as Hypothalamic-Pituitary-Adrenal axis dysregulation, resulting in elevated cortisol levels and systemic inflammation, ultimately leading to increased CVD risk^10^.

Structural discrimination can also confirm stereotypes, which can alter community norms and social interactions, leading to the occurrence of interpersonal discrimination events^11^. Interpersonal discrimination is defined as unfair treatment expressed through interactions between people based on biases against social identities^14^. Examples of interpersonal discrimination include but are not limited to workplace discrimination (e.g., hiring discrimination) and everyday microaggressions (e.g., assumptions of criminality). Perceiving and appraising interpersonal discrimination events can prompt negative emotional responses, which at a chronic level, can ultimately lead to physiological decline and the development of chronic health conditions like CVD^11^.

Previous systematic reviews explored the impact of psychosocial factors, including race-related stressors, on pregnancy-related and cardiometabolic outcomes in women. However, systematic reviews examining outcomes during pregnancy are sparse^15^. They also fail to include cardiometabolic outcomes, such as gestational diabetes, and instead emphasize offspring outcomes such as preterm birth or low birth weight^16–19^. Additionally, some reviews did not test key race-related stressors, such as perceived discrimination^20^ and to our knowledge, none of these reviews to date have examined the link between perceived neighborhood stressors and pregnancy-related or cardiometabolic outcomes in women. Perceived stressors, such as perceived neighborhood stressors, capture an individual’s subjective appraisal and emotional response to their neighborhood environments, which can then directly impact health outcomes^21,22^. On the other hand, objective measures assess the presence of physical attributes of a neighborhood but do not capture how these attributes are internalized or their associated psychological impact, which play a key role in contributing to health outcomes through physiological stress-response pathways^21^.

The objective of this systematic review was to provide an overview of the current literature quantifying the impact of perceived neighborhood stressors and discrimination (PNSD) on gestational diabetes and hypertensive disorders, which are two major cardiometabolic outcomes in pregnant women in the US. This systematic review specifically (1) identified and collected relevant studies, (2) examined the reported associations between PNSD and cardiometabolic outcomes during pregnancy, and how these associations varied across racial and/or ethnic groups, and (3) evaluated the methodological quality and potential biases of the included studies. The overarching goals of this study were to evaluate the current literature on PNSD and cardiometabolic outcomes during pregnancy, determine the extent and quality of the evidence, and identify future research directions.

## Results

### Study selection

**Fig. 1:**
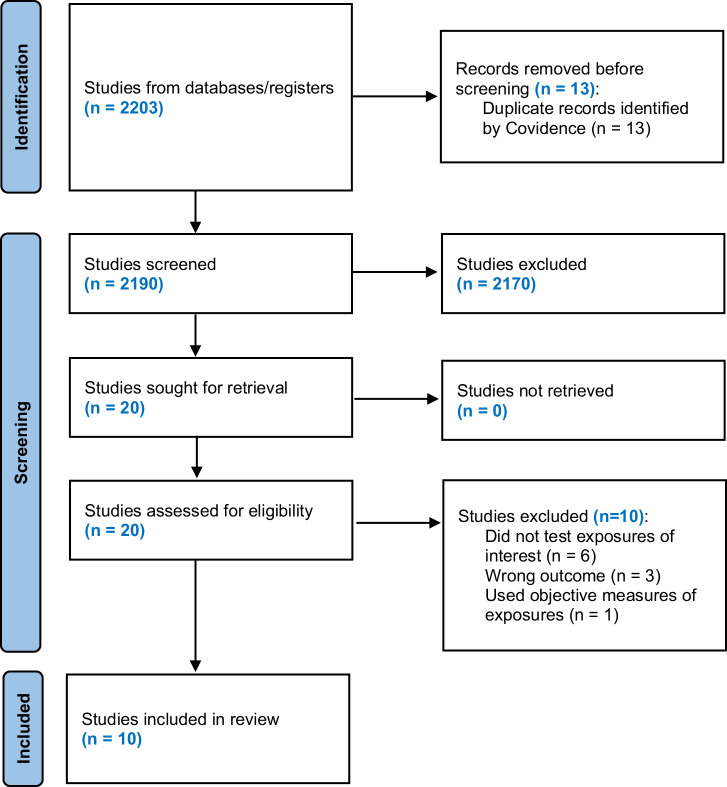
Preferred Reporting Items for Systematic Reviews and Meta-Analyses (PRISMA) flowchart. The PRISMA flowchart for the selection of studies that assessed PNSD and cardiometabolic outcomes during pregnancy.

The initial search identified 2203 potentially relevant articles, which were reduced to 2190 articles after excluding 13 duplicate records. During title and abstract screening, 2170 studies were excluded due to irrelevance or failure to meet inclusion/exclusion criteria. This exclusion resulted in 20 articles eligible for full-text review, of which 10 articles were excluded for the following reasons: six studies did not explicitly include PNSD as the study exposure, three studies did not assess gestational diabetes or hypertensive disorders of pregnancy as the study outcome(s), and one study examined objective rather than perceived neighborhood stressors. In total, 10 studies were included in the systematic review (Fig. 1).

### Study characteristics

Seven out of 10 articles employed a longitudinal cohort study design, whereas the remaining studies (*n* = 3) used a cross-sectional design (Table 1). The sample sizes ranged from 204 to 9907 participants. Four studies were located within a single geographical location, with two of these studies in New York^23,24^, one study in Georgia^25^, and one study in Wisconsin^26^. Two studies used a study population with predominantly Black participants^25,27^ and only one study included a population exclusively composed of Black women^26^.

**Table 1:** Characteristics of selected studies

Author and publication year	Study characteristics			Participant characteristics			Race-Related Stressor	Cardiometabolic Outcome During Pregnancy	Key Findings
	Study design	Data source	Sample size	Age^a^ Mean (SD)	Race and/or Ethnicity^b^	Geographic Location			
Caplan, 2021	Cohort study	Measurement of maternal stress study	647	• HDP group: 29.68 (2.25) • No HDP group: 29.39 (5.70)	HDP group • White: 56.5% • Black: 9.4% • Hispanic: 29.4% • Other: 4.7% No HDP group • White: 61.0% • Black: 16.5% • Hispanic: 18.5% • Other: 4.0%	Four clinical sites across the United States	Perceived discrimination (Williams’ Discrimination Survey)^c^	Development of hypertensive disorders of pregnancy	There was no association between perceived discrimination and HDP risk (OR = 1.16, 95% CI: 0.94–1.44).
Erbetta, 2022	Cohort study	2012–2014 New York City Pregnancy Risk Assessment Monitoring System	4084	• <25: 22.3% • 25-34: 54.1% • 35+: 23.5%	US Born • White: 20.7% • Black: 11.8% • Hispanic: 12.5% • Asian/Pacific Islander/Other: 2.6% Foreign-Born • White: 8.5% • Black: 9.3% • Hispanic: 20.1% • Asian/Pacific Islander/Other: 14.2%	New York City	Perceived racial discrimination	Gestational diabetes	Racial discrimination was associated with an increased risk of gestational diabetes in unadjusted models (RR = 1.57, 95% CI: 1.19–2.06). This was no longer significant in fully adjusted models (RR = 1.24, 95% CI: 0.87–1.78).
Grobman, 2018	Cohort study	Nulliparous pregnancy outcomes study: monitoring mothers-to-be	9470	Not available	• White: 60.4% • Black: 13.8% • Hispanic: 16.7% • Asian: 4.0% • Other: 5.0%	Participants recruited from 8 hospitals around the nation	Perceived racism (Krieger Racism Scale)	Development of hypertensive disorders of pregnancy	There was no association between perceived racism and HDP risk (OR = 0.81, 95% CI: 0.62–1.06).
Joseph, 2023	Cohort study	Clinic patients	1608	27.7 (6.4)	• Hispanic: 12.1% • White: 2.1% • Black: 82.2% • Other: 3.6%	Georgia	Perceived housing insecurity	• Preeclampsia without severe features • Preeclampsia with severe features • Gestational diabetes	Perceived housing insecurity was significantly associated with a lower risk of preeclampsia without severe features (RR = 0.75, 95% CI: 0.63–0.89).
Lawson, 1999	Cross-sectional	Convenience sample	323	46.2 (2.2)	• Black: 91.6% • White: 0.3% • Multicultural: 8.1%	United States	Perceived racial discrimination	Pregnancy-related problems (including hypertension)	Experiencing racial discrimination was associated with increased pregnancy-related problems (*r* = 0.27, *p* < 0.01).
Lee, 2024	Cohort study	Pregnancy Risk Assessment Monitoring System phase 8 (2016–2020)	9907	• 17–19: 4.7% • 20–24: 17.5% • 25–29: 27.5% • 30–34: 30.4% • 35–39: 15.9% • 40 + : 3.9%	• Hispanic Non-White: n=832 • Hispanic White: n=967 • American Indian & Hawaiian: n=281 • Asian: n=808 • Black: n=2697 • Non-White or mixed race: n=874 • White: n=3448	17 states across the United States	Perceived racism and/or perceived discrimination	Development of hypertensive disorders of pregnancy	There was no association between perceived racism or discrimination and HDP risk (OR = 0.94, 95% CI: 0.74–1.20). When stratifying across racial and/or ethnic group, perceived discrimination was associated with a lower risk of hypertensive disorders of pregnancy among Asian participants only.
MacGregor, 2020	Cohort study	Measurement of maternal stress study	595	• Participants who developed Gestational diabetes: 31.8 (5.4) • Participants who did not develop Gestational diabetes: 29.6 (5.5)	Participants who developed Gestational diabetes • White: 58.0% • Black: 16.0% • Hispanic: 24.0% • Other: 2.0% Participants who did not develop Gestational diabetes • White: 62.2% • Black: 16.7% • Hispanic: 14.9% • Other: 6.2%	Four clinical sites across the United States	Perceived discrimination (Williams’ Discrimination Survey)^c^	Gestational diabetes	Perceived discrimination was associated with increased risk of developing gestational diabetes (OR = 2.11; 95% CI, 1.03–4.22).
Sharma, 2022	Cross-sectional study	National Health Interview Survey 2013–2017	1433	28.8 (5.5)	• White: n=778 • Black: n=216 • Asian: n=93 • Hispanic: n=321	United States	Created a composite SDOH score utilizing a domain for “neighborhood, physical environment, and social cohesion”	• Hypertension during pregnancy • Suboptimal CVD defined as ≥2 cardiovascular risk factors including hypertension and diabetes	Higher SDOH score was associated with greater risk for suboptimal CVD (RR = 2.05, 95% CI: 1.46–2.88).
Tannis, 2018	Cohort study	Clinic patients	379	• <20 years: 9.4% • ≥20 years: 90.6%	• Black/African-American: 27.7% • Hispanic/Latina: 68.6% • Other: 3.7%	East Harlem, New York	• Perceived neighborhood safety • Perceived poor housing quality	Preeclampsia	In unadjusted models, poor housing quality was significantly associated with a lower odds of preeclampsia (OR = 0.50, 95% CI: 0.28–0.89). This association was no longer significant in fully adjusted models (OR = 0.67, 95% CI: 0.32–1.38).
Walker, 2024	Cross-sectional study	Clinic-based recruitment of patients within the Froedtert Health System, Community-based recruitment	204	30.9 (5.6)	Black: 100%	Milwaukee, Wisconsin	• Perceived discrimination (Experiences of Discrimination Scale) • Perceived neighborhood crime • Perceived neighborhood violence	Preeclampsia	Perceived neighborhood crime was significantly associated with a greater risk of preeclampsia (OR = 1.72, 95% CI: 1.05–2.83).

*HDP* hypertensive disorders of pregnancy; *SD* standard deviation, *SDOH* social determinants of health, *CVD* cardiovascular disease.

^a^Age is reported as “Mean (SD)” unless the authors of the study provided “%”.

^b^Studies reported “%” for racial and/or ethnic participant breakdown, unless indicated with “*n*” if the study reported number of participants.

^c^“Williams’ Discrimination Survey,” as referred to in the studies, is a less common name that authors of the studies used to refer to the Everyday Discrimination Scale.

### Perceived Neighborhood Stressors and Discrimination (PNSD) measures

Of the two race-related stressors of interest (PNSD), four studies only focused on perceived neighborhood stressors^24,25,28^, six studies focused solely on perceived discrimination^23,27,29–32^, and one study examined both PNSD^26^.

Among the four studies that assessed perceived neighborhood stressors, one study focused on perceived neighborhood insecurity^25^, another on perceived neighborhood safety and housing quality^24^, and another on perceived neighborhood crime and violence^26^. One study used a composite SDOH score with six domains, including a domain for neighborhood physical environment and social cohesion^28^ (Table 1).

The discrimination scales used were the “Williams’ Discrimination Survey,” commonly known as the Everyday Discrimination Scale, to assess perceived everyday discrimination (*n* = 2)^29,32^, as well as the Krieger Racism Scale (*n* = 1)^31^ and the Experiences of Discrimination scale (*n* = 1)^26^ to assess perceived racial discrimination. The remaining three studies that assessed perceived discrimination used a single-item measure to assess if participants had ever experienced racial discrimination in the past 12 months^23,27,30^ (Table 1).

### Cardiometabolic outcomes during pregnancy

Five studies assessed the development of hypertensive disorders of pregnancy exclusively^24,26,30–32^, two studies solely assessed the development of gestational diabetes^23,29^, and one study examined both hypertensive disorders of pregnancy and gestational diabetes^25^. Additionally, one study included hypertensive disorders of pregnancy in a broad category of “pregnancy-related problems”^27^, and another study assessed cross-sectional associations with the presence of hypertension and diabetes in participants interviewed during pregnancy^28^. Most studies assessed cardiometabolic outcomes during pregnancy through record linkages (e.g., electronic health records, state-based surveillance systems) (*n* = 7), while the rest of the articles (*n* = 3) assessed cardiometabolic outcomes during pregnancy via participant self-report.

### Examination of associations between perceived neighborhood stressors and cardiometabolic outcomes during pregnancy

Four studies tested the direct association between perceived neighborhood stressors and the development of hypertensive disorders of pregnancy^24–26,28^ (Table 1). One study reported that greater perceived housing insecurity was associated with a lower risk of preeclampsia without severe features^25^, and another documented that poor housing quality was associated with lower odds of preeclampsia in unadjusted analyses only^24^. Conversely, only one study indicated a significant positive association, such that higher perceived neighborhood crime was associated with a higher risk of preeclampsia^26^. In contrast, perceived neighborhood safety and violence were not related to preeclampsia risk^24,26^. Additionally, a composite SDOH score, including a singular domain for neighborhood physical environment and social cohesion constructs, was not associated with hypertension during pregnancy^28^.

Only one study assessed the link between perceived neighborhood stressors, specifically perceived housing insecurity, and gestational diabetes, but did not find an association^25^ (Table 1). Another study reported an association between a composite SDOH score, including neighborhood physical environment and social cohesion, with subclinical CVD risk (based on factors including hypertension and diabetes during pregnancy)^28^.

### Examination of associations between perceived discrimination and cardiometabolic outcomes during pregnancy

Five studies tested the association between perceived discrimination and the development of hypertensive disorders of pregnancy ^26,27,30–32^ (Table 1). Only one of these studies reported an association between perceived racial discrimination and increased risk of pregnancy-related problems, including hypertensive disorders of pregnancy^27^. The remaining four studies, which examined hypertensive disorders of pregnancy as a separate outcome, reported null associations.

Two studies examined the association between perceived discrimination and gestational diabetes^23,29^. One study focused on racial discrimination using a single-item measure and only found an association in unadjusted models^23^, whereas another study assessed everyday discrimination and found an association in fully adjusted models^29^.

### Racial and/or ethnic differences

Only one of these ten studies examined how the association between PNSD and cardiometabolic outcomes during pregnancy may differ across racial and/or ethnic groups. Specifically, one study tested the association between perceived discrimination and hypertensive disorders of pregnancy stratified by racial and/or ethnic group and found an association between perceived discrimination and lower risk of hypertensive disorders of pregnancy among Asian participants only^30^. However, there were null associations between perceived discrimination and hypertensive disorders of pregnancy across all other racial and/or ethnic groups^30^.

### Risk of bias assessment

Most studies in this systematic review were of good methodological quality, based on the Newcastle-Ottawa Scale (NOS) (Tables 2 and 3). Cohort studies had a mean NOS score of 0.89^23–25,29–32^. The “Selection” domain of the NOS was the most common risk of bias among cohort studies, particularly due to recruiting participants from a single geographical location, which limits the exposed cohort’s representativeness to the national population of pregnant women^23–25^.

**Table 2:** Quality assessment of included cohort studies

Author and publication year	Exposure	Outcome	Key finding	Selection (Max 4 points)	Comparability (Max 2 points)	Outcome (Max 3 points)	Total score (Max 9 points)
Caplan, 2021	Perceived discrimination (Williams’ Discrimination Survey)^a^	Development of hypertensive disorders of pregnancy	No association	4	2	3	9
Erbetta, 2022	Perceived racial discrimination	Gestational diabetes	Racial discrimination was associated with an increased risk of gestational diabetes in unadjusted models	3	2	3	8
Grobman, 2018	Perceived racism (Krieger Racism Scale)	Development of hypertensive disorders of pregnancy	No association	4	2	3	9
Joseph, 2023	Perceived discrimination (Williams’ Discrimination Survey)^a^	Gestational diabetes	Perceived discrimination was associated with increased risk of developing gestational diabetes	3	2	3	8
Lee, 2024	Perceived racism and/or perceived discrimination	Development of hypertensive disorders of pregnancy	No association	2	2	2	6
MacGregor, 2020	Perceived discrimination (Williams’ Discrimination Survey)^a^	Gestational diabetes	Perceived discrimination was associated with increased risk of developing gestational diabetes	4	2	3	9
Tannis, 2018	Perceived neighborhood safety, Perceived poor housing quality	Preeclampsia	In unadjusted models, poor housing quality was significantly associated with a lower odds of preeclampsia	3	1	3	7

^a^“Williams’ Discrimination Survey,” as referred to in the studies, is a less common name that authors of the studies used to refer to the Everyday Discrimination Scale.

**Table 3:** Quality assessment of included cross-sectional studies

Author and publication year	Exposure	Outcome	Key finding	Selection (Max 3 points)	Comparability (Max 2 points)	Outcome (Max 3 points)	Total score (Max 8 points)
Lawson, 1999	Perceived racial discrimination	Pregnancy-related problems (including hypertension)	Experiencing racial discrimination was associated with increased pregnancy-related problems	0	1	2	3
Sharma, 2022	Composite SDOH score	Hypertension during pregnancy; Suboptimal CVD	Higher SDOH score was associated with greater risk for suboptimal CVD	3	2	2	7
Walker, 2024	Perceived discrimination (Experiences of Discrimination Scale), Perceived neighborhood crime, Perceived neighborhood violence	Preeclampsia	Perceived neighborhood crime was significantly associated with a greater risk of preeclampsia	2	2	3	7

*SDOH* social determinants of health, *CVD* cardiovascular disease.

Cross-sectional studies had a mean NOS score of 0.71^26–28^. The most common risk of biases for cross-sectional studies included assessment of the cardiometabolic outcome using self-report rather than record linkages (e.g., electronic health records, state-level surveillance systems)^27,28^ and small sample sizes^26,27^. All cohort studies, except for one that used self-reported measures^30^, received the maximum points in the “Outcome” domain. Across both cross-sectional and cohort studies, the “Comparability” domain had the lowest risk of bias overall, with strong group comparability in most studies except for two studies which did not sufficiently control for potential confounding factors^24,27^.

## Discussion

This is the first systematic review to our knowledge that examined the associations between PNSD and cardiometabolic outcomes during pregnancy (hypertensive disorders of pregnancy and gestational diabetes). This systematic review of ten studies found no demonstrable associations between PNSD and hypertensive disorders of pregnancy. However, the limited research suggests an association between PNSD and an increased risk of gestational diabetes. Overall, the studies included in this systematic review demonstrated good methodological quality.

Among the five studies that examined perceived neighborhood stressors, each focused on a different dimension of neighborhood stressors. One study reported a significant positive association between perceived neighborhood crime and increased risk of preeclampsia^26^, while another linked a composite SDOH score (including a single domain for neighborhood physical environment and social cohesion) to subclinical CVD and CVD risk factors, though the composite score prevented the isolation of the specific impact of neighborhood stressors^28^. These findings are consistent with previous studies that reported associations between neighborhood stressors and cardiometabolic outcomes in the general population^33^, as well as other adverse pregnancy outcomes (e.g., preterm birth)^34^. However, other studies in this review either reported null associations^24–26,28^ or negative associations such that higher perceived neighborhood stressors were associated with a lower risk of preeclampsia^24,25^. This pattern is also consistent with findings from several studies using objective measures of neighborhood environment, where green spaces and walkability were inversely associated or not associated with cardiometabolic outcomes during pregnancy^35^. It is also important to note that each study in the current review focused on different neighborhood stressors: perceived neighborhood insecurity^25^, perceived neighborhood safety and housing quality^24^, perceived neighborhood crime and violence^26^, and a single domain for neighborhood physical environment and social cohesion within a broader SDOH score^28^. Given that each stressor was examined in only one study, there is insufficient evidence to draw reliable conclusions about the association between each neighborhood stressor (e.g., perceived neighborhood insecurity, neighborhood safety, housing quality, neighborhood crime, neighborhood violence) and cardiometabolic outcomes during pregnancy. Two of the studies investigating perceived neighborhood stressors were also limited by small sample sizes (*N* = 204 and *N* = 379), which reduced statistical power^24,26^. Additionally, three of these five studies were conducted at a single site^24–26^, which is a significant limitation since neighborhood perceptions are highly location-dependent.

Perceived discrimination was consistently associated with increased risk for gestational diabetes^23,29^. However, this relationship is likely driven by multiple pathways, as one of these two studies tested obesity as a mediator but found that obesity accounted for less than a quarter of the association^29^. It is also important to note that only two studies investigated this direct association. Additionally, among the four studies that examined the relationship between perceived discrimination and hypertensive disorders of pregnancy as an isolated outcome, no significant associations were found^26,30–32^. This finding was unexpected given previous systematic reviews consistently linking perceived discrimination to cardiometabolic outcomes in the general population^36^, as well as with non-cardiometabolic pregnancy outcomes (e.g., preterm birth)^19^. One explanation may be that two of the included studies assessed if and/or how often participants reported experiencing discrimination over the past year, which is a limited time frame^30,32^. This narrow approach fails to capture the cumulative effects of lifetime discrimination, which can result in a greater impact over time on pregnancy outcomes. Some studies also reported low endorsement of discrimination among participants due to excluding non-English speakers^32^ or not administering questionnaires in certain states in the US^30^, which could then limit the ability to detect a meaningful association between discrimination and cardiometabolic outcomes during pregnancy. Another potential explanation for this finding may be that two of the four studies had relatively small sample sizes (*N* = 204 and *N* = 647), which reduced the statistical power to detect statistically significant associations. Each of these studies also used a different scale to measure discrimination, with most questionnaires assessing discrimination *only* attributed to race (e.g., Krieger Racism Scale, Experiences of Discrimination scale, Pregnancy Risk Assessment Monitoring System racial discrimination item), highlighting the need for standardized measures to allow for comparison. This approach also overlooked the intersectionality of discrimination, as pregnant women may face compounded discrimination due to race *along with* other marginalized identities, such as sex, age, or physical appearance.

A summary of recommendations for future research (Table 4) was developed as a result of this systematic review. Larger study populations are needed in future studies to investigate these relationships. More research is also needed to understand how these associations may differ across racial and/or ethnic groups. Only one study tested these racial and/or ethnic differences, finding that perceived discrimination was associated with a lower risk of hypertensive disorders of pregnancy among Asian participants, suggesting a protective effect for this group^30^. However, no studies examined how the associations between perceived discrimination and gestational diabetes, as well as the associations between perceived neighborhood stressors and any cardiometabolic outcome during pregnancy may differ across racial and/or ethnic groups. Individuals within each racial and/or ethnic group may experience these stressors differently because, for example, racial residential segregation may lead to different neighborhood perceptions^37^. Furthermore, the broad categorizations of racial and/or ethnic groups used by most studies may mask heterogeneity within these groups. Racial and/or ethnic populations underrepresented in research, such as American Indian or Alaska Native, Native Hawaiian or Pacific Islander groups, are frequently aggregated into one broad category “Other”^23–25,29–32^ or excluded from studies altogether. More research needs to be conducted to include these underrepresented racial and/or ethnic groups, as well as examine intra-group heterogeneity of perceived psychosocial stressors and cardiometabolic conditions. Additionally, there is a need to identify the various mechanisms (e.g., biological disruptions to hypothalamic–pituitary–adrenal axis, behavioral responses such as diet and physical activity, engagement with the healthcare system) through which PNSD affects cardiometabolic outcomes during pregnancy. Conducting a future meta-analysis would also be helpful for synthesizing and analyzing data across studies and determining the overall effect size for these associations. Future studies could also recruit participants from multiple geographical locations to capture a broad range of neighborhood perceptions, which are location-dependent. This systematic review only included studies with participants in the US. Due to the unique historical context that contributed to discrimination and neighborhood stressors^38,39^, and because of the different definitions of neighborhoods used across countries^40,41^, future research focused on cross-country comparisons could examine the impact of discrimination and neighborhood stressors on pregnancy outcomes across countries. There is also a critical gap in examining *perceived* neighborhood stressors and the impact on cardiometabolic outcomes during pregnancy, as evidenced by the small number of studies in this review. The few prior existing systematic reviews that examined neighborhood stressors^16,20^ relied on objective measures of neighborhood stressors, such as Census data for neighborhood poverty or local police records for neighborhood crime. However, these objective measures fail to capture individuals’ unique experiences and their perceptions of systemic barriers^22^. To our knowledge, no studies tested whether perceived neighborhood stressors (e.g., social disorder, social cohesion) were associated with cardiometabolic outcomes during pregnancy, and thus, research needs to be conducted on all forms of perceived neighborhood stressors. Future studies could also include both perceived and objective measures to detect stress at biological levels in those who may have been desensitized to neighborhood stressors. Additionally, research focusing on perceived discrimination could utilize questionnaires that better capture the intersectional nature of discrimination.

**Table 4:** Future research recommendations

Race-related stressors of interest	Recommendations
Both stressors (neighborhood stressors and discrimination)	• Utilize a larger study population to increase statistical power • Test how associations differ by racial and/or ethnic groups • Research the various mechanisms through which these associations manifest • Conduct a meta-analysis to analyze data from multiple studies and confirm associations
Neighborhood stressors	• Recruit participants from multiple geographical locations, including in other countries • Research perceived neighborhood stressors, as well as in conjunction with objective neighborhood stressors • Examine other neighborhood stressors (e.g., perceived neighborhood social disorder)
Discrimination	• Develop and validate questionnaires that consider the intersectionality of discrimination experienced by pregnant women

Future research recommendations for studies investigating the association between perceived neighborhood stressors or discrimination with cardiometabolic outcomes during pregnancy.

The quality assessment revealed several risks of biases across the included studies (e.g., small sample sizes, single-site recruitment, and ascertainment of outcome via self-report rather than record linkage). The use of study populations with small sample sizes reduced statistical power, which could explain the null findings observed^24,26,27,29,32^. Four of the ten included studies recruited participants from a single site, or geographical location^23–26^. Single-site recruitment, such as one study recruiting participants from the same clinic in East Harlem, New York^24^, may limit generalizability of the findings to pregnant women in the US, especially because neighborhood perceptions and health outcomes can significantly vary across locations within the US. Additionally, single-site recruitment may also lead to selection bias as the associations may only reflect the specific geographical location from which all the participants were drawn. Three studies utilized questionnaires through which participants self-reported having hypertensive disorders of pregnancy or gestational diabetes^27,28,30^. Some of these studies found null associations between PNSD and hypertensive disorders of pregnancy^28,30^. Recall and social desirability biases due to self-reporting health outcomes may be one potential explanation for these findings, since participants could inaccurately recall or feel pressured to underreport health outcomes. Additionally, three of the ten studies were cross-sectional, which prevented the assessment of temporality (e.g., whether preeclampsia developed before or after perceiving high levels of neighborhood crime)^26–28^. This makes causal inference difficult, as it is unclear whether the cardiometabolic outcomes were due to PNSD, or if the conditions themselves may have played a role in the perceptions of these stressors. However, one strength of almost all included studies was their comparability because controlling for multiple confounding variables (e.g., age, education) improved robustness of the associations.

Limitations should be noted when interpreting findings from this systematic review. The inclusion criteria restricted studies to English-language articles, which may result in publication bias. Additionally, several of the included studies used small sample sizes, which could have contributed to the null associations identified in this systematic review due to reduced statistical power, which presents challenges for detecting accurate effects. Many studies also recruited participants from a single site or geographical location, which reduced the generalizability of findings to the target population. Furthermore, this systematic review only included studies published in peer-reviewed journals and excluded grey literature (e.g., dissertations, conference abstracts) to increase the probability that studies would be of high methodological quality and include valid findings. However, this decision may have introduced publication bias since findings such as null or inverse associations are less likely to be published in peer-reviewed journals^42^.

Despite these limitations, this systematic review had several strengths. This review addressed a knowledge gap regarding the synthesis of the recent literature on PNSD and cardiometabolic outcomes during pregnancy. This review also highlighted how these associations may vary across racial and/or ethnic groups. An exhaustive search of the literature was conducted using several databases (PubMed, PsycINFO, Embase, Web of Science, and CINAHL). Additionally, using Covidence allowed for a more organized and streamlined screening process that was less prone to errors. This systematic review focused on perceived rather than objective neighborhood stressors, which provided more insight into how subjective experiences of race-related stressors, which are typically more relevant to individual behaviors and perceptions, could impact cardiometabolic outcomes during pregnancy. Finally, the detailed list of recommendations for future research outlined in this systematic review helped to identify existing gaps in the literature that need to be addressed to improve our understanding of the relationship between PNSD and cardiometabolic outcomes during pregnancy.

To our knowledge, this systematic review is the first to synthesize the literature on the relationship between perceived race-related stressors, specifically PNSD, and cardiometabolic outcomes during pregnancy. Although this systematic review revealed largely null findings for the relationship between PNSD and hypertensive disorders of pregnancy, there is some evidence to support an association between perceived discrimination and increased risk for gestational diabetes. This systematic review addressed several knowledge gaps, but additional research is warranted to further understand the impact of various forms of perceived neighborhood stressors on cardiometabolic outcomes during pregnancy. Understanding that PNSD contributes to cardiometabolic outcomes during pregnancy can help healthcare professionals identify at-risk patient populations, allowing for timely screening and interventions to mitigate the health effects of PNSD.

## Methods

The methodology and reporting in this review followed the Preferred Reporting Items for Systematic Reviews and Meta-Analysis (PRISMA) guidelines for a systematic review without a meta-analysis (see Supplementary Materials). The study protocol was registered in Open Science Framework on October 16, 2024, and additional details can be found at 10.17605/OSF.IO/DX3PR.

### Exposures and outcomes of interest

The primary exposures of interest in this systematic review were PNSD. Perceived neighborhood stressors refer to an individual’s perceptions of their neighborhood’s physical and social environment. Discrimination is defined as experiencing unfair treatment based on one or more marginalized social identities. Both perceived measures were assessed using self-report measures, which capture an individual’s subjective experiences and awareness of stressors.

The primary outcomes of interest in this systematic review were the incidence or prevalence of cardiometabolic outcomes during pregnancy, specifically hypertensive disorders of pregnancy and gestational diabetes.

### Search strategy

The search strategy was developed by a trained librarian at the National Institutes of Health and included a combination of keywords, synonyms, and Medical Subject Headings (MeSH) terms related to hypertensive disorders of pregnancy, gestational diabetes, pregnancy, PNSD, and racial and/or ethnic minority groups. The databases were searched on September 24, 2024. The following electronic databases were searched for articles published any time before September 24, 2024: MEDLINE via PubMed (National Library of Medicine), PsycINFO (American Psychological Association), Embase (Elsevier), Web of Science Core Collection (Clarivate Analytics), and CINAHL (EBSCO). The citation lists of all included studies were also searched to identify any additional relevant studies. The retrieved articles from the search were saved in Covidence, a web-based tool used to screen studies when conducting systematic reviews, and all duplicate articles were deleted from Covidence (www.covidence.org).

### Inclusion and exclusion criteria

The following pre-determined criteria were used to select articles that would be included in the systematic review: (1) published in English-language peer-reviewed journals at any time before September 24, 2024, (2) examined either perceived neighborhood stressors or discrimination and the association with hypertension or gestational diabetes during pregnancy, (3) studied pregnant women ≥18 years of age, and (4) studied participants living in the US. Animal studies, literature reviews, case reports, conference proceedings, clinical trials, pre-prints, and dissertations were excluded from the systematic review.

### Study screening and selection

Each article was independently screened in Covidence by two reviewers (RD, undisclosed reviewer [UR]) who used the inclusion/exclusion criteria to assess eligibility for article selection in the systemic review. Each reviewer assessed the content in the titles and abstracts, and then performed a more in-depth full-text review of the relevant articles. All conflicts during the screening process were resolved by a third and fourth reviewer (KT, ATF).

### Data extraction

After the screening and selection processes were completed, two extractors (RD, UR) collected the following information in Covidence from each of the selected articles included in the systematic review: author, year of publication, study design, data source, sample size, participant sociodemographic characteristics, race-related stressors, cardiometabolic outcomes during pregnancy, key findings, and quality/potential biases.

### Quality assessment

Two independent reviewers (RD, UR) applied the Newcastle-Ottawa scale adapted for cohort and cross-sectional studies (see Supplementary Materials) to assess the quality and potential risk of bias in the articles selected for review (http://www.ohri.ca/programs/clinical_epidemiology/oxford.asp). This scale assessed observational studies for bias using three domains: selection of study population, comparability of groups, and determining exposure and outcome variables. The Newcastle-Ottawa scale had high inter-rater reliability^43^ and required less time per study to complete compared to other similarly purposed assessment tools^44^. Regarding cohort studies, the scale included eight items, and a maximum of one point could be assigned to each of the eight items, except for the “Compatibility” domain which could be assigned a maximum of two points. However, for cross-sectional studies, there were seven items in the scale, and a maximum of one point could be assigned to each of the items except for the “Assessment of Outcome” item, which could receive up to two points. Therefore, each cohort and cross-sectional study could receive a maximum of nine and eight points, respectively. Reviewers used these scales to assess the quality of all included studies, and any disagreements between reviewers on the quality assessments were resolved through discussion.

## Supplementary information

Supplementary Information

## Data Availability

Data sharing is not applicable to this article as no datasets were generated or analyzed during the current study.
