# Supplementary material for: A systematic review of the relationship between neighborhood stressors, discrimination, and cardiometabolic outcomes during pregnancy
Source: NPJ Womens Health. 2025 Apr 25;3(1):25. doi: 10.1038/s44294-025-00072-0 (PMC12031668; doi:10.1038/s44294-025-00072-0)
Supplement: Supplementary file 1 — Supplementary Information [file 44294_2025_72_MOESM1_ESM.pdf]

A systematic review of the relationship between neighborhood stressors, discrimination and cardiometabolic outcomes during pregnancy

Supplementary Materials

Newcastle-Ottawa Scale for Cohort Studies

SELECTION: 1) Representativeness of the exposed cohort

SELECTION: A study can be awarded a maximum of one star for each numbered item within the Selection

a) truly representative of the average _______________ (describe) in the community [One Point]

b) somewhat representative of the average ______________ in the community [One Point]

c) selected group of users e.g. nurses, volunteers

d) no description of the derivation of the cohort

SELECTION: 2) Selection of the non exposed cohort

SELECTION: A study can be awarded a maximum of one star for each numbered item within the Selection

a) drawn from the same community as the exposed cohort [One Point]

b) drawn from a different source

c) no description of the derivation of the non exposed cohort

SELECTION: 3) Ascertainment of exposure

SELECTION: A study can be awarded a maximum of one star for each numbered item within the Selection

a) secure record (e.g. surgical records) [One Point]

b) structured interview [One Point]

c) written self report

d) no description

SELECTION: 4) Demonstration that outcome of interest was not present at start of study

SELECTION: A study can be awarded a maximum of one star for each numbered item within the Selection

a) Yes [One Point]

b) No

COMPARABILITY: Comparability of cohorts on the basis of the design or analysis

COMPARABILITY: A maximum of two stars can be given for Comparability

a) study controls for _____________ (select the most important factor) [One Point]

b) study controls for any additional factor [One Point](This criteria could be modified to indicate specific control for a second important factor.)

c) No

OUTCOME: 1) Assessment of outcome

OUTCOME: A study can be awarded a maximum of one star for each numbered item within the Outcome

a) independent blind assessment [One Point]

b) record linkage [One Point]

c) self report

d) no description

OUTCOME: 2) Was follow-up long enough for outcomes to occur

OUTCOME: A study can be awarded a maximum of one star for each numbered item within the Outcome

a) yes (select an adequate follow up period for outcome of interest) [One Point]

b) no

OUTCOME: 3) Adequacy of follow up of cohorts

OUTCOME: A study can be awarded a maximum of one star for each numbered item within the Outcome

a) complete follow up - all subjects accounted for [One Point]

b) subjects lost to follow up unlikely to introduce bias - small number lost - > ____ % (select an adequate %) follow up, or description provided of those lost) [One Point]

c) follow up rate < ____% (select an adequate %) and no description of those lost

d) no statement

Newcastle-Ottawa Scale for Cross-Sectional Studies

SELECTION: 1) Representativeness of the sample

CROSS SECTION STUDIES SELECTION part - (Maximum 3 points/stars)

a) if the sample was truly representative of the average in the target population (all subjects or random sampling) or somewhat representative (non-random sampling) [One Point]

b) no description of included subjects.

SELECTION: 2) Sample size

CROSS SECTION STUDIES SELECTION part - (Maximum 3 points/stars)

a) if sample size was justified and satisfactory [One Point]

b) no information provided

SELECTION: 3) Not Included Subjects

CROSS SECTION STUDIES SELECTION part - (Maximum 3 points/stars)

a) if comparability between included and non-included subjects was established, and if the inclusion rate was satisfactory. [One Point]

b) no comparability between included and non-included subjects was established

COMPARABILITY: 1a) Subjects in different outcome groups are comparable, based on study design or analysis. Confounding factors are controlled.

CROSS SECTION STUDIES COMPARABILITY part - (Maximum 2 points/stars)

a) if there was adequate adjustment of anthropometric measures for age and gender. [One Point]

b) information was not provided or groups were not comparable.

COMPARABILITY: 1b) Subjects in different outcome groups are comparable, based on study design or analysis. Confounding factors are controlled.

CROSS SECTION STUDIES COMPARABILITY part - (Maximum 2 points/stars)

a) if there was adequate adjustment for other factors influencing the outcome, such as race/ethnicity, poverty-to-income ratio, educational level and health behavior. [One Point]

b) information was not provided or groups were not comparable.

OUTCOME: 1) Assessment of outcome

CROSS SECTION STUDIES OUTCOME part -(Maximum 2 points/stars)

a) if outcomes were assessed independently and blindly or from record linkage (database records) [Two Point]

b) if the outcome was assessed by self-report [One Point]

c) none of the above

OUTCOME: 2) Statistical test

CROSS SECTION STUDIES OUTCOME part -(Maximum 1 point/stars)

a) if the statistical test used to analyze the data was clearly described and appropriate, and the measurement of the association was presented, including confidence intervals and the probability level (p value). [One Point]

b) no - the statistical test used to analyze the data was not clearly described

PRISMA Checklist

Section and Topic	Item #	Checklist item	Location where item is reported
**TITLE**			
Title	1	Identify the report as a systematic review.	Title, Line 1
**ABSTRACT**			
Abstract	2	See the PRISMA 2020 for Abstracts checklist.	Abstract, Page 2
**INTRODUCTION**			
Rationale	3	Describe the rationale for the review in the context of existing knowledge.	Introduction, Pages 3-4
Objectives	4	Provide an explicit statement of the objective(s) or question(s) the review addresses.	Introduction, Lines 86-95
**METHODS**			
Eligibility criteria	5	Specify the inclusion and exclusion criteria for the review and how studies were grouped for the syntheses.	Methods, Lines 389-396
Information sources	6	Specify all databases, registers, websites, organisations, reference lists and other sources searched or consulted to identify studies. Specify the date when each source was last searched or consulted.	Methods, Lines 380-383
Search strategy	7	Present the full search strategies for all databases, registers and websites, including any filters and limits used.	Methods, Lines 376-379
Selection process	8	Specify the methods used to decide whether a study met the inclusion criteria of the review, including how many reviewers screened each record and each report retrieved, whether they worked independently, and if applicable, details of automation tools used in the process.	Methods, Lines 398-403
Data collection process	9	Specify the methods used to collect data from reports, including how many reviewers collected data from each report, whether they worked independently, any processes for obtaining or confirming data from study investigators, and if applicable, details of automation tools used in the process.	Methods, Lines 364-373, 405-410

Section and Topic	Item #	Checklist item	Location where item is reported
Data items	10a	List and define all outcomes for which data were sought. Specify whether all results that were compatible with each outcome domain in each study were sought (e.g. for all measures, time points, analyses), and if not, the methods used to decide which results to collect.	Methods, Lines 364-373, 405-410
	10b	List and define all other variables for which data were sought (e.g. participant and intervention characteristics, funding sources). Describe any assumptions made about any missing or unclear information.	Methods, Lines 405-410
Study risk of bias assessment	11	Specify the methods used to assess risk of bias in the included studies, including details of the tool(s) used, how many reviewers assessed each study and whether they worked independently, and if applicable, details of automation tools used in the process.	Methods, Lines 412-428
Effect measures	12	Specify for each outcome the effect measure(s) (e.g. risk ratio, mean difference) used in the synthesis or presentation of results.	Not applicable.
Synthesis methods	13a	Describe the processes used to decide which studies were eligible for each synthesis (e.g. tabulating the study intervention characteristics and comparing against the planned groups for each synthesis (item #5)).	Methods, Lines 405-410
	13b	Describe any methods required to prepare the data for presentation or synthesis, such as handling of missing summary statistics, or data conversions.	Not applicable. No statistics required.
	13c	Describe any methods used to tabulate or visually display results of individual studies and syntheses.	Methods, Lines 405-410
	13d	Describe any methods used to synthesize results and provide a rationale for the choice(s). If meta-analysis was performed, describe the model(s), method(s) to identify the presence and extent of statistical heterogeneity, and software package(s) used.	Methods, Lines 405-410
	13e	Describe any methods used to explore possible causes of heterogeneity among study results (e.g. subgroup analysis, meta-regression).	Not applicable.
	13f	Describe any sensitivity analyses conducted to assess robustness of the synthesized	Not applicable.

Section and Topic	Item #	Checklist item	Location where item is reported
		results.	
Reporting bias assessment	14	Describe any methods used to assess risk of bias due to missing results in a synthesis (arising from reporting biases).	Methods, Lines 412-428
Certainty assessment	15	Describe any methods used to assess certainty (or confidence) in the body of evidence for an outcome.	Methods, Lines 412-428
**RESULTS**			
Study selection	16a	Describe the results of the search and selection process, from the number of records identified in the search to the number of studies included in the review, ideally using a flow diagram.	Results, Lines 98-106
	16b	Cite studies that might appear to meet the inclusion criteria, but which were excluded, and explain why they were excluded.	Results, Lines 98-106
Study characteristics	17	Cite each included study and present its characteristics.	Results, Lines 108-141, Table 1
Risk of bias in studies	18	Present assessments of risk of bias for each included study.	Results, Lines 182-186, Tables 2 and 3
Results of individual studies	19	For all outcomes, present, for each study: (a) summary statistics for each group (where appropriate) and (b) an effect estimate and its precision (e.g. confidence/credible interval), ideally using structured tables or plots.	Results, Table 1
Results of syntheses	20a	For each synthesis, briefly summarise the characteristics and risk of bias among contributing studies.	Results, Lines 182-186, Tables 2 and 3
	20b	Present results of all statistical syntheses conducted. If meta-analysis was done, present for each the summary estimate and its precision (e.g. confidence/credible interval) and measures of statistical heterogeneity. If comparing groups, describe the direction of the effect.	Not applicable.
	20c	Present results of all investigations of possible causes of heterogeneity among study	Not applicable.

Section and Topic	Item #	Checklist item	Location where item is reported
		results.	
	20d	Present results of all sensitivity analyses conducted to assess the robustness of the synthesized results.	Not applicable.
Reporting biases	21	Present assessments of risk of bias due to missing results (arising from reporting biases) for each synthesis assessed.	Results, Lines 182-186, Tables 2 and 3
Certainty of evidence	22	Present assessments of certainty (or confidence) in the body of evidence for each outcome assessed.	Results, Lines 182-186, Tables 2 and 3
**DISCUSSION**			
Discussion	23a	Provide a general interpretation of the results in the context of other evidence.	Discussion section, Lines 198-355
	23b	Discuss any limitations of the evidence included in the review.	Discussion section, Lines 198-355
	23c	Discuss any limitations of the review processes used.	Discussion section, Lines 198-355
	23d	Discuss implications of the results for practice, policy, and future research.	Discussion section Lines 256-297, 345-355
**OTHER INFORMATION**			
Registration and protocol	24a	Provide registration information for the review, including register name and registration number, or state that the review was not registered.	Methods, Lines 360-362
	24b	Indicate where the review protocol can be accessed, or state that a protocol was not prepared.	Methods, Lines 360-362
	24c	Describe and explain any amendments to information provided at registration or in the protocol.	Not applicable.
Support	25	Describe sources of financial or non-financial support for the review, and the role of the funders or sponsors in the review.	Acknowledgements Lines 430-435

Section and Topic	Item #	Checklist item	Location where item is reported
Competing interests	26	Declare any competing interests of review authors.	Lines 445-446
Availability of data, code and other materials	27	Report which of the following are publicly available and where they can be found: template data collection forms; data extracted from included studies; data used for all analyses; analytic code; any other materials used in the review.	Lines 448-450

From: Page MJ, McKenzie JE, Bossuyt PM, Boutron I, Hoffmann TC, Mulrow CD, et al. The PRISMA 2020 statement: an updated guideline for reporting systematic reviews. BMJ 2021;372:n71. doi: 10.1136/bmj.n71. This work is licensed under CC BY 4.0. To view a copy of this license, visit https://creativecommons.org/licenses/by/4.0/
